# Germanium-doped Metallic Ohmic Contacts in Black Phosphorus Field-Effect Transistors with Ultra-low Contact Resistance

**DOI:** 10.1038/s41598-017-16845-w

**Published:** 2017-12-04

**Authors:** Hsun-Ming Chang, Adam Charnas, Yu-Ming Lin, Peide D. Ye, Chih-I Wu, Chao-Hsin Wu

**Affiliations:** 1 Graduate Institute of Photonics and Optoelectronics, National Taiwan University, No. 1, Sec. 4, Roosevelt Road, Taipei, 10617 Taiwan (R.O.C.); 2 Graduate Institute of Electronics Engineering, National Taiwan University, No. 1, Sec. 4, Roosevelt Road, Taipei, 10617 Taiwan (R.O.C.); 3 Taiwan Semiconductor Manufacturing Company No. 8, Li-Hsin Rd. 6, Hsinchu Science Park, Hsinchu City, 300 Taiwan (R.O.C.); 4 Industrial Technology Research Institute, 195, Sec. 4, Chung Hsing Rd., Chutung Hsinchu City, 31040 Taiwan (R.O.C.); 5 School of Electrical and Computer Engineering and Birck Nanotechnology Center, Purdue University, West Lafayette Indiana, 47907 United States

## Abstract

In this work, we demonstrate for the first time an ultra-low contact resistance few-layered black phosphorus (BP) transistor with metallic PGe_x_ contacts formed by rapid thermal annealing (RTA). The on-state current of the transistor can be significantly improved and the I_ON_/I_OFF_ ratio increases by almost 2 order. The hole mobility is enhanced by 25 times to 227 cm^2^V^−1^s^−1^. The contact resistance extracted by the transfer length method is 0.365 kΩ∙μm, which is the lowest value in black phosphorus transistors without degradation of I_ON_/I_OFF_ ratio. In addition, the I-V curve of the transistor with PGe_x_ contact is linear compared to that with Ti contact at 80 K, indicating that a metallic ohmic contact is successfully formed. Finally, X-ray photoelectron spectroscopy is used to characterize the PGe_x_ compound. A signal of P-Ge bond is first observed, further verifying the doping of Ge into BP and the formation of the PGe_x_ alloy.

## Introduction

Two-dimensional (2D) materials have shown great potential in next-generation electronic applications. The scaling of state-of-the-art transistors has demanded device dimensions in the sub-10 nm range^1^. However, traditional silicon-based metal-oxide-semiconductor field-effect transistors (MOSFETs) face severe problems of short channel effects as devices scale down^2^. In order to maintain proper gate control for channel electrostatics, one of the solutions is using ultra-thin channel body to reduce the geometric screening length Λ = ((ε_ch_/Nε_ox_) t_ox_ t_ch_)^0.5^, where t_ox_ and t_ch_ are the oxide thickness and the channel thickness, respectively; ε_ox_ and ε_ch_ are the relative permittivities of oxide and channel materials, respectively^3^. Nevertheless, traditional bulk semiconductors such as Si and III-V materials suffer from the issue of mobility degradation while the body thickness scales down to nanoscale order^4–7^. On the other hand, 2D materials have attracted a large amount of interest due to their superior material properties. Layered 2D materials consist of covalent intralayer bonding of each atom and van der Waals (vdW) interlayer interactions. Taking advantage of their layered structure, the desirable properties of ultra-thin body and atomically smooth interfaces without surface roughness scattering can exist simultaneously^8,9^. The first discovered and most widely studied 2D material is graphene^10^. In spite of the high carrier mobility and thermal conductivity of graphene, the lack of band gap energy limits its further application to logic circuits^11^. The family of transition metal dichalcogenides (TMDs) such as MoS_2_ offers larger band gaps, but the relatively low carrier mobility restrains the output performance of field effect transistors (FETs)^12–14^. Recently, black phosphorus (BP) was found to be a promising 2D semiconductor for high-performance transistor devices^15^. BP is the most stable allotrope of phosphorus, and the bulk material can be synthesized from white phosphorus or red phosphorus under high pressure and high temperature^16,17^. BP has a tunable direct band gap, ranging from 0.3 eV for bulk to 1.5–2 eV for monolayer^18,19^. In addition, BP owns a high carrier mobility beyond 1000 cm^2^V^−1^s^−1^ at room temperature^18,20^. The above properties fill the gap between graphene and TMDs in terms of bandgap and carrier mobility, and make BP an attractive candidate for high-performance electronics as well as other optoelectronics applications^21^. For example, a BP radio-frequency transistor has recently been reported with a cut-off frequency (f_T_) up to 12 GHz^22^. In spite of the great material properties, the crucial issue that restrains the output performance of BP is the large contact resistance (R_c_) in the interface of source/drain metal and BP channel. Compared to 3D (bulk) materials, the pristine surfaces of a 2D material tends to form by van der Waals (vdW) gap with contact metal instead of covalent bonds^23^. This vdW gap acts as an additional tunneling barrier for channel carriers to enter the metal contact, thus resulting in a higher contact resistance. So far, the most adopted strategy to reduce contact resistance is by choosing different work functions of metals. For example, a high work function metal such as palladium (Pd) allows more hole carriers to inject into the contact, hence improving the contact resistance for p-type BP transistors^24–26^. On the contrary, a relatively low work function metal like aluminum (Al) improves the electron carrier injection and leads to n-type behavior in BP^26,27^. However, this method cannot solve the problems of interfacial vdW gap. Another strategy is by the method of surface-charge-transfer doping of electrophilic molecules in channel region^28,29^. Although surface-charge-transfer doping can provide a relative low R_c_ (0.66 kΩ∙μm for the record low value), I_ON_/I_OFF_ ratio of the device is sacrificed in most cases. Besides, some of the doping effects exhibit poor long-term air-stability^30^. Therefore, a new method needs to be developed to reduce contact resistance by forming metallic alloy between contact metal and BP.

In the previous research, germanium (Ge) is doped into bulk BP by mixing Ge into BP powder during bismuth-flux preparation^31^. As a group IV element, Ge acts as an acceptor when doping into BP, which is a group V element. The results show that the resistivity of forming PGe_x_ compound is reduced by 2–3 orders and its temperature dependence exhibits metallic properties even at low temperature^31^. Based upon this concept, we apply a group IV element, e.g. Ge, as the source/drain metal to reduce the contact resistivity in BP transistors. In this work, we present for the first time a few-layered BP FET with germanium-doped metallic contact. We successfully dope germanium into the contact region of black phosphorus through rapid thermal annealing process (RTA). The electrical characteristics including I_ON_/I_OFF_ ratio, mobility, on-state current density, on-state resistance, and contact resistance are analyzed for devices before and after annealing. Finally, X-ray photoelectron spectroscopy (XPS) analysis is carried out to characterize the formation of germanium-doped black phosphorus.

## Results and Discussion

**Figure 1:**
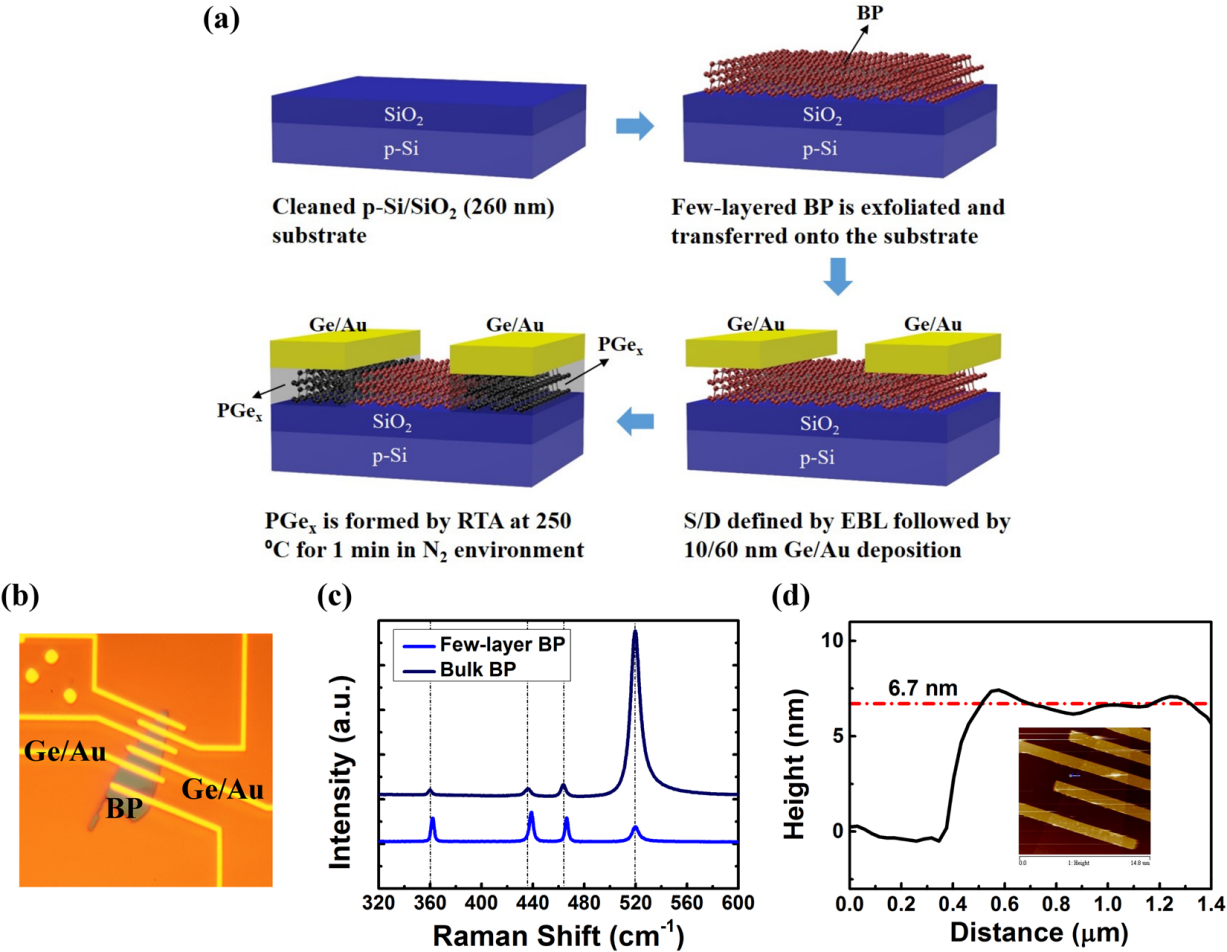
(**a**) Schematic fabrication process flow of a PGe_x_-contact BP transistor. (**b**) Optical microscopy picture of the BP transistor. (**c**) Raman spectroscopy of the BP transistor (**d**) The thickness of the same device measured by AFM.

Few-layered BP flakes are first mechanically exfoliated from bulk crystal and then transferred onto silicon substrates with 260 nm thermally grown SiO_2_ using a polydimethylsiloxane (PDMS) stamp^32^. The exfoliation and transfer processes are executed inside a glove box flowed with nitrogen to reduce moisture absorption and oxidation of BP. Source/drain contact bars with 1.1 µm contact length are defined through standard electron beam lithography (EBL) process. A metal stack of 10 nm Ge followed by 60 nm Au are deposited for metal contacts by electron-beam thermal evaporator to complete a back-gate BP transistor. The few-layered BP transistor is loaded into a vacuum environment (<10^−2^ Torr) immediately after device fabrication for electrical characteristics measurement. After the preliminary measurement, the device is then transferred to RTA equipment. The chamber is pumped down to vacuum and filled with nitrogen at first, and then heated up to 250 °C rapidly and kept for 1 minute. After RTA treatment, the annealed device is electrically characterized in the vacuum environment again. The schematic process flow of device fabrication is illustrated in Fig. 1a. After RTA treatment, germanium will be doped into BP under contact region, forming PGe_x_ compound. In the later part of this article, we will discuss about the RTA-treated Ge contact and verify the formation of a metallic PGe_x_ contact. The optical microscopy picture of the device is shown in Fig. 1b. The results of Raman spectroscopy of the few-layered BP transistor and bulk BP as reference are shown in Fig. 1c, where the peak at 520 cm^-1^ refers to the signal of Si substrate. In bulk BP, there exhibits three peaks at 359.75 cm^−1^, 436.17 cm^−1^ and 463.71 cm^−1^, which corresponds to A^1^_g_, B^2^_g_, and A^2^_g_ phonon modes due to the lattice vibration of BP^33^. However, as the thickness scales down to the few-layered flake, a red-shift of three characteristic peaks to 361.93 cm^−1^, 438.64 cm^−1^ and 466.43 cm^−1^ is observed since the oscillation of P atoms increases as thickness decreases^34^. Moreover, the Raman peaks of the few-layered BP transistor indicates its thickness is pretty thin^34^. As shown in Fig. 1d, the average thickness of the BP flake is 6.7 nm measured by atomic force microscopy.

**Figure 2:**
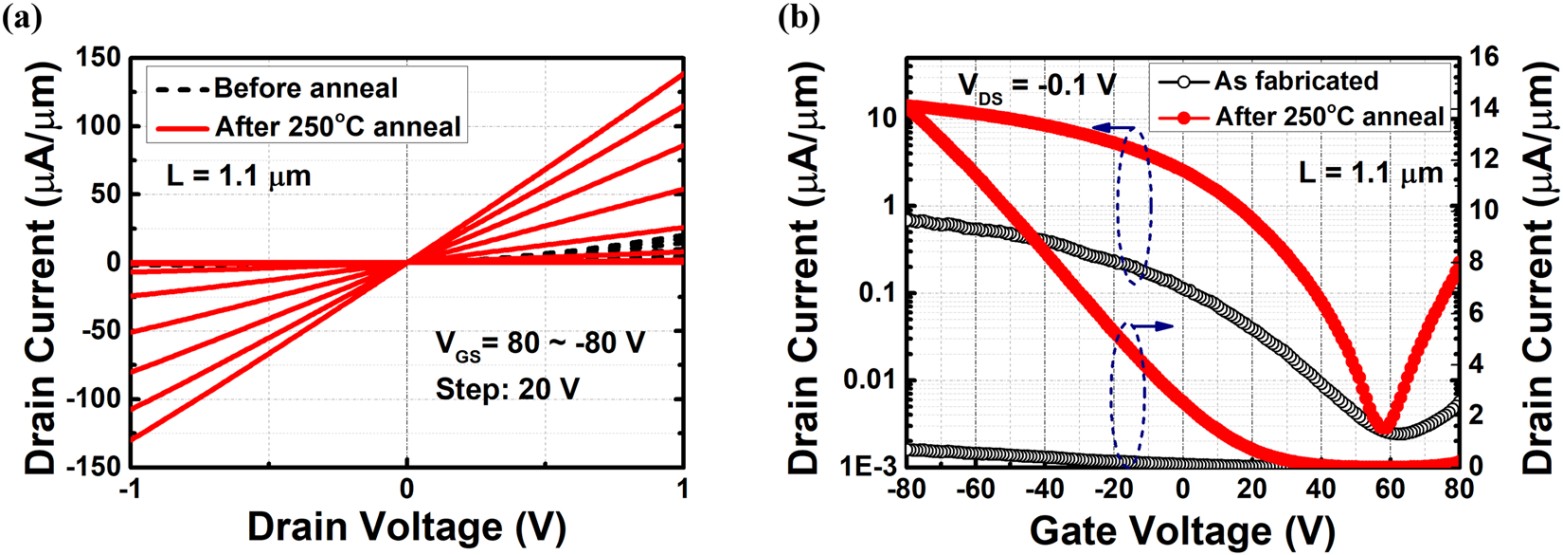
(**a**) Output characteristics and (**b**) Transfer curve of a Ge contact BP transistor before and after 250 °C rapid thermal annealing. Transfer characteristics are shown in both semilogarithmic scale and linear scale.

Figure 2 compares the transfer and output characteristics of the device with 1 µm channel before and after RTA treatment, which correspond to the black and red curves in the figure, respectively. The output characteristics before and after RTA treatment is demonstrated in Fig. 2a, with a back gate voltage sweeps from +80 V to −80 V. The output characteristics of as-prepared device shows non-linear Schottky contacts with asymmetric I_DS_-V_DS_ behaviors at positive and negative V_DS_, indicating a poor contact of germanium on BP before annealing. However, the output current is greatly enhanced after 250 °C annealing. The on-state current density, which is defined as the drain current density at an overdrive voltage (V_GS_ − V_TH_) of −40 V and V_DS_ of −1 V, is improved by 4.84 times (from 14.46 µA/µm to 70.07 µA/µm). In this work, V_TH_ is obtained by linear extrapolation of I_DS_ to the intercept of V_GS_. In addition, the annealed device shows linear I_DS_-V_DS_ characteristics, indicating the formation of Ohmic contact. Figure 2b demonstrates the transfer characteristics at drain voltage of −0.1 V and the applied back gate voltage sweeping from +80 V to −80 V in both semilogarithmic and linear scale. After 250 °C annealing, the I_ON_/I_OFF_ ratio shown in semilogarithmic scale increases from 10^2^ ~ 10^3^ to almost 10^4^, which can be attributed to the enhancement of on current after annealing since both minimum currents remain unchanged. From the transfer curve in linear scale we can calculate the extrinsic effective mobility in the linear region by μ_eff_ = G_m_L/(C_ox_WV_DS_), where C_ox_ is the oxide capacitance, W and L are the channel width and channel length, and G_m_ is the peak transconductance. After annealing, the extrinsic hole mobility μ_eff_ is 127.9 cm^2^V^−1^s^−1^, which is 21 times higher than the value of 5.9 cm^2^V^−1^s^−1^ before RTA. The annealing does not cause the shift of the minimum current point, indicating that the channel doping is not affecting by the annealing at contact. The significant boost of the effective mobility is attributed to the following two reasons: the reduction of contact resistance, and the improvement of channel interfaces. When contacts improve from Schottky to Ohmic, the current injection into metals is greatly enhanced. In addition, annealing also improves the interfaces of BP channel. BP tends to absorb the moisture in the air, resulting in charge trap impurities and degradation of mobility^35^. Annealing helps remove the moisture in both BP surface and BP/SiO_2_ interface, thus enhancing the mobility^36^. The improvement of the interfaces can be further verified by the reduction of hysteresis in the transfer curve shown in Figure S1, which results from defects and impurities at the channel interfaces. Nevertheless, the hysteresis is still large compared to ref.^36^ after annealing. The reason is probably that although moisture absorption is removed, a large amount of fixed charges in the thick oxide and BP/SiO_2_ interfacial traps still exist^25^.

**Figure 3:**
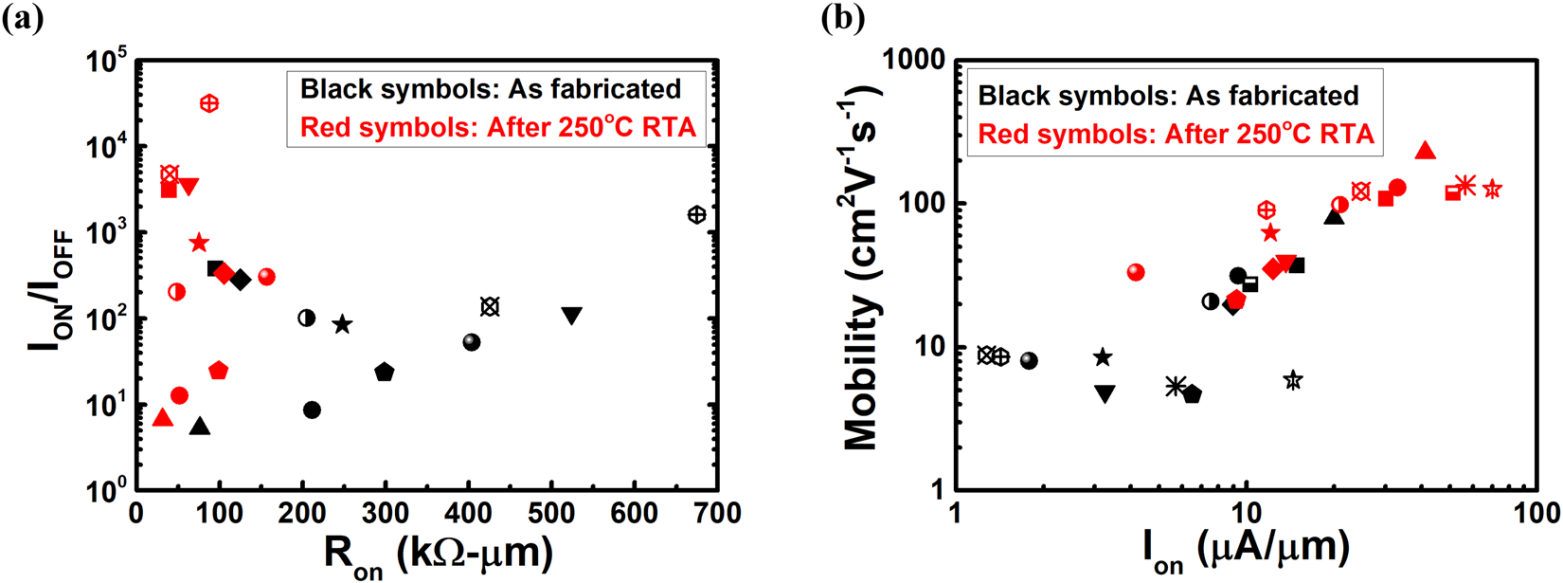
(**a**) I_ON_/I_OFF_ versus R_ON_ before and after annealing of various devices. After annealing, I_ON_/I_OFF_ increases and R_ON_ decreases. (**b**) Hole mobility versus I_ON_ before and after annealing of various devices. After RTA treatment, hole mobility and I_ON_ both increase, with the maximum mobility of 227 cm^2^V^−1^s^−1^.

More than one BP device with Ge contact are fabricated using the same fabrication process. The electrical characteristics of these devices including I_ON_, R_ON_, mobility, and I_ON_/I_OFF_ ratio before and after RTA are summarized in Fig. 3. A common trend of increasing I_ON_/I_OFF_ ratio and reducing R_ON_ after RTA can be observed in Fig. 3a. In addition, both mobility and I_ON_ improve after RTA, as shown in Fig. 3b. The maximum mobility of 227 cm^2^V^−1^s^−1^ is readily achieved with the maximum improvement of mobility of 25.1 times. These results show the consistent enhancement of output characteristics using Ge as the metal contact with RTA process. On the other hand, a titanium (Ti) contact BP FET is also fabricated for comparison with the same fabrication process except that the S/D metal contact is replaced by 10 nm Ti followed by 60 nm Au. The electrical results are presented in Figure S2. As shown in Figure S2a, the output characteristics and the non-linearity of the drain current show no improvement after RTA. R_ON_ slightly rises from 85.6 to 88.7 kΩ∙μm after annealing. In Figure S2b, the transfer curve clearly shows a left-shift of V_TH_ and a more ambipolar characteristics after annealing. The extrinsic hole mobility decreases from 68.6 to 66 cm^2^V^−1^s^−1^ after annealing. In fact, most of our devices with Ti contact share the same trend after annealing: V_TH_ shifts left, R_ON_ increases, and hole mobility decreases. The possible reasons may be due to the tendency of oxidization of Ti^37^. Although annealing can improve interfaces of BP channel, the oxidation of Ti degrades S/D contacts and restrains the overall output performance.

**Figure 4:**
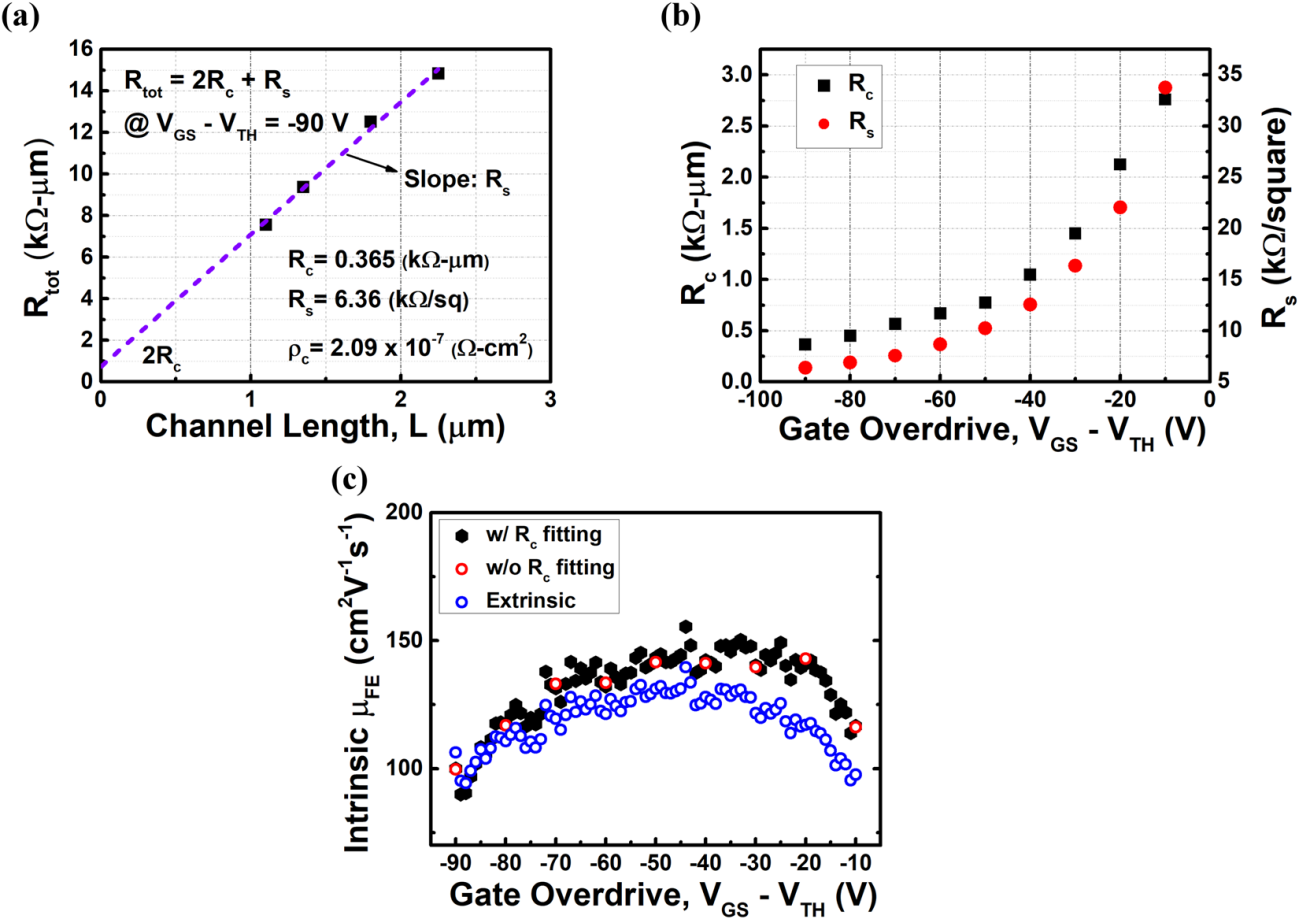
(**a**) TLM results of a BP transistor at gate overdrive voltage of −90 V. A contact resistance (R_c_) of 1.18 kΩ∙μm can be obtained by extrapolation to L = 0. (**b**) Contact resistance (R_c_) and sheet resistance (R_s_) versus various gate overdrive voltages. As gate overdrive voltage becomes more negative (p-type doping), both R_c_ and R_s_ decrease. (**c**) Intrinsic and extrinsic mobility versus gate overdrive voltage. Blue circles represents extrinsic mobility, while red and black dots represent the intrinsic mobility extracted from experimental R_c_ and from the fitting results of R_c_, respectively.

The contact resistance (R_c_) of the device after RTA treatment is extracted from the TLM pattern by two-probe measurement, as shown in Fig. 4a. R_c_ can be obtained by extrapolation of the total resistance (R_tot_) to zero channel length (L). The extracted contact resistance is 0.365 kΩ∙μm at gate overdrive voltage of −90 V. To our knowledge, this is the lowest reported contact resistance for BP devices and is even comparable to that for III-V devices^28,38,39^. Moreover, instead of sacrificing I_ON_/I_OFF_ ratio suffered from surface transfer doping, this approach of Ge-doped contact can further improve the I_ON_/I_OFF_ ratio as mentioned above. Figure 4b demonstrates the relationship of R_c_ and R_s_ versus the gate overdrive voltage. As the overdrive becomes more negative, the BP channel will be more electrostatically doped with more induced hole carriers. Consequently, contact resistance and sheet resistance both decrease. To calculate the intrinsic mobility, μ_int_, the following equation ${I}_{DS}={\mu }_{int}{C}_{ox}\frac{W}{L}({V}_{GS}-{V}_{TH}-{I}_{DS}{R}_{c})({V}_{DS}-2{I}_{DS}{R}_{c})$ is applied. Similar to the extraction of extrinsic mobility, we first calculate G_m_ and extract the intrinsic mobility from it. Note that R_c_ is also dependent on gate voltage; hence dR_c_/dV_GS_ needs to be considered. The details of μ_int_ extraction is mentioned in the supplementary information. The extraction results are shown in Fig. 4c, where the extrinsic mobility corresponds to blue circles, while red and black dots represent the intrinsic mobility extracted from experimental R_c_ and from the fitting results of R_c_, respectively. A maximum μ_int_ of about 150 cm^2^V^−1^s^−1^ can be obtained in this method.

A more detailed investigation on the RTA-treated Ge contact is discussed in the following. The results of the Ti contact BP transistor mentioned above are also analyzed for comparison. In order to characterize the contact properties of Ti and RTA-treated Ge contact, a low temperature measurement is performed. In the traditional metal-semiconductor junction theory, there are two mechanisms of carrier injection in a metal-semiconductor barrier: thermionic emission (TE) and field emission (FE, or tunneling)^40^. The basic transport equation in a metal-semiconductor junction is given by J = J_SM_ − J_MS_, where J is the total current density, J_SM_ and J_MS_ is the current density flowing from semiconductor to metal and metal to semiconductor, respectively. The specific contact resistance can be defined as

1 $${R}_{c}\equiv {(\frac{\partial J}{\partial V})}_{V=0}^{-1}$$

where J is the total current density in the junction as mentioned above. When the junction is dominated by thermionic emission, which is also the major transport mechanism of Schottky contacts, the total current density is given by

2 $$J={A}^{\ast }{T}^{n}{e}^{-\frac{q{\varphi }_{B}}{kT}}({e}^{\frac{qV}{kT}}-1)$$

where ${A}^{\ast }$ is the Richardson constant, *ϕ*_B_ is the Schottky barrier height, *k* is Boltzmann constant and *n* is an exponent equal to 2 for bulk semiconductors and 3/2 for 2D semiconductors^23^. From Eq. (1) and Eq. (2), the specific contact resistance dominant by thermionic emission can be obtained as

3 $${R}_{c}=\frac{k}{q{A}^{\ast }{T}^{0.5}}{e}^{\frac{q{\varphi }_{B}}{kT}}$$

**Figure 5:**
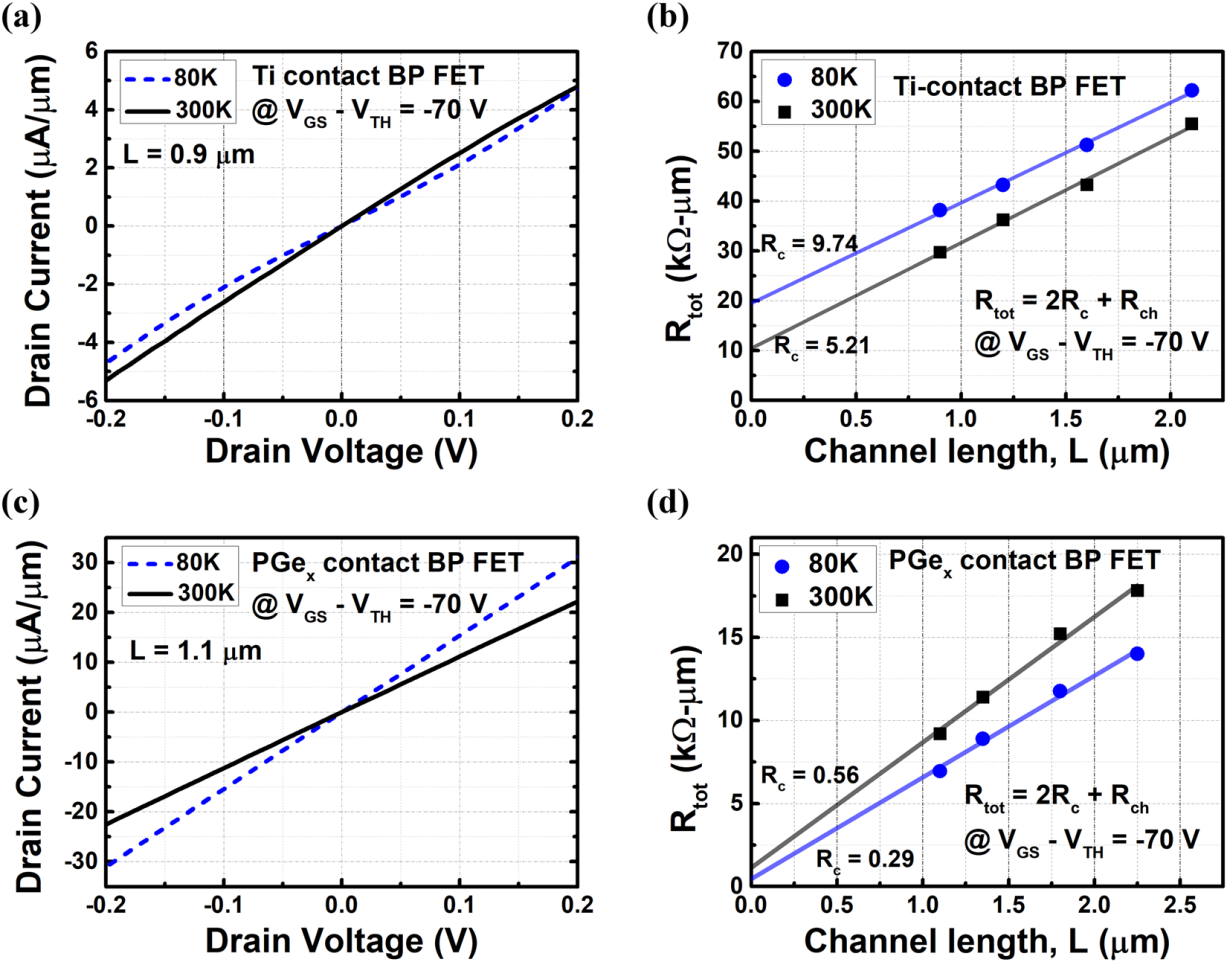
(**a**) Output characteristics of a Ti-contact BP transistor at 80 K and 300 K with gate overdrive voltage of −70 V. The I-V curve becomes non-linear Schottky-like from 300 K to 80 K. (**b**) TLM results of a Ti-contact BP transistor at 80 K and 300 K with gate overdrive voltage of −70 V. R_c_ increases from 5.21 to 9.74 kΩ∙μm as temperature decreases from 300 to 80 K, which is the characteristics dominated by thermionic emission. (**c**) Output characteristics of a PGe_x_-contact BP transistor at 80 K and 300 K with gate overdrive voltage of −70 V. Drain current varies linearly with drain voltage as temperature decrease to 80 K. (**d**) TLM results of a PGe_x_-contact BP transistor at 80 K and 300 K with gate overdrive voltage of −70 V. As temperature decreases from 300 K to 80 K, R_c_ decreases from 1.55 to 0.87 kΩ∙μm, indicating a metallic contact is formed.

It is clear from Eq. (3) that the contact resistance decreases exponentially as temperature increases. On the other hand, when field emission (or tunneling) dominates the current transport, which is often the case of a highly doping Ohmic contact, *R*_*c*_ can be calculated using WKB approximation and is given by^40^

4 $${R}_{c} \sim (\frac{1}{{E}_{00}}){e}^{\frac{q{V}_{b0}}{{E}_{00}}}$$

where *V*_*b*0_ is the built-in potential and ${E}_{00}=\frac{\hslash }{2}\sqrt{\frac{N}{{{\epsilon }}_{s}{m}^{\ast }}}$ is the characteristic energy in which *N* is the doping concentration, *∈*_*S*_ is the permittivity of semiconductor, and *m* ^*^ is the effective mass. From Eq. (4), *R*_*c*_ is independent of temperature. In a more accurate derivation, *R*_*c*_ will decrease slightly as temperature increase and become less dependent on temperature as the doping concentration increases^41,42^. Therefore, we can examine the dominant transport mechanism at the contact by characterizing the behavior of *R*_*c*_ between low and high temperature. Figure 5 displays the I-V curve and TLM results at room temperature (300 K) and 80 K for both Ti contact and RTA-treated Ge contact. All of the results are measured at the same gate overdrive voltage of −70 V. In Fig. 5a and b, the I-V curves and TLM results of Ti contact are shown, respectively. At 300 K, the drain current varies almost linearly with drain voltage. However, the I-V curves at 80 K shows clearly the behavior of a Schottky contact, where drain current slowly increases at lower drain voltage and then increases faster when drain voltage further drives up. In addition, the extracted *R*_*c*_ of 9.74 kΩ∙μm at 80 K is higher than that (5.21 kΩ∙μm) at 300 K, further showing the properties of thermionic emission. The sheet resistance of 20.16 kΩ/sq at 80 K is lower than that (21.19 kΩ/sq) at 300 K due to less phonon scattering at low temperature. It is clear from the results that the transport mechanism at Ti-BP contact is composed of a certain extent of thermionic emission. Note that although the Ti-contact device behaves as an “Ohmic-like” contact at 300 K, the thermionic transport is still non-negligible even at an electrostatically doping of −70 V by gate, which means it is an “Ohmic-like” Schottky contact instead of a “real” Ohmic contact. On the other hand, the I-V curves and TLM results of PGe_x_ contact are shown in Fig. 5c and d, respectively. Different from Ti contact, the I_DS_-V_DS_ curves exhibit good linearity even at 80 K for PGe_x_ contact. This indicates that thermionic emission is negligible even at low temperature. Moreover, the slope at 80 K is higher than at 300 K, which means a lower R_ON_ is obtained at 80 K. As shown in the TLM results, the lower contact resistance of 0.29 kΩ∙μm and sheet resistance of 6.12 kΩ/sq at 80 K both contribute to the lower R_ON_ at 80 K. The decrease of R_s_ from 7.55 to 6.12 kΩ/sq is also due to the less scattering effect at lower temperature similar to the case of Ti-contact. However, the increase of R_c_ from 0.29 to 0.56 kΩ∙μm as temperature increases demonstrates the contrary trend of field emission transport. Theoretically, R_c_ will be slightly dependent on temperature or almost independent of temperature in field emission transport. To analyze this interesting behavior, we fit the temperature-dependent contact resistivity (${\rho }_{c}$) by power law of ${\rho }_{c}(T)\propto {T}^{\gamma }$ in Figure S4, where the power index, ${\rm{\gamma }}$, of +3.8 is obtained. The results show that the contact displays a typical behavior of “metals,” since the resistivity of metals follow the power law of ${\rho }_{c}(T)\propto {T}^{\gamma }$ with 1 < γ < 5^43^. It is for the first time that a metallic contact of BP transistors is demonstrated, which potentially overcomes the bottleneck of contact issue in BP devices.

**Figure 6:**
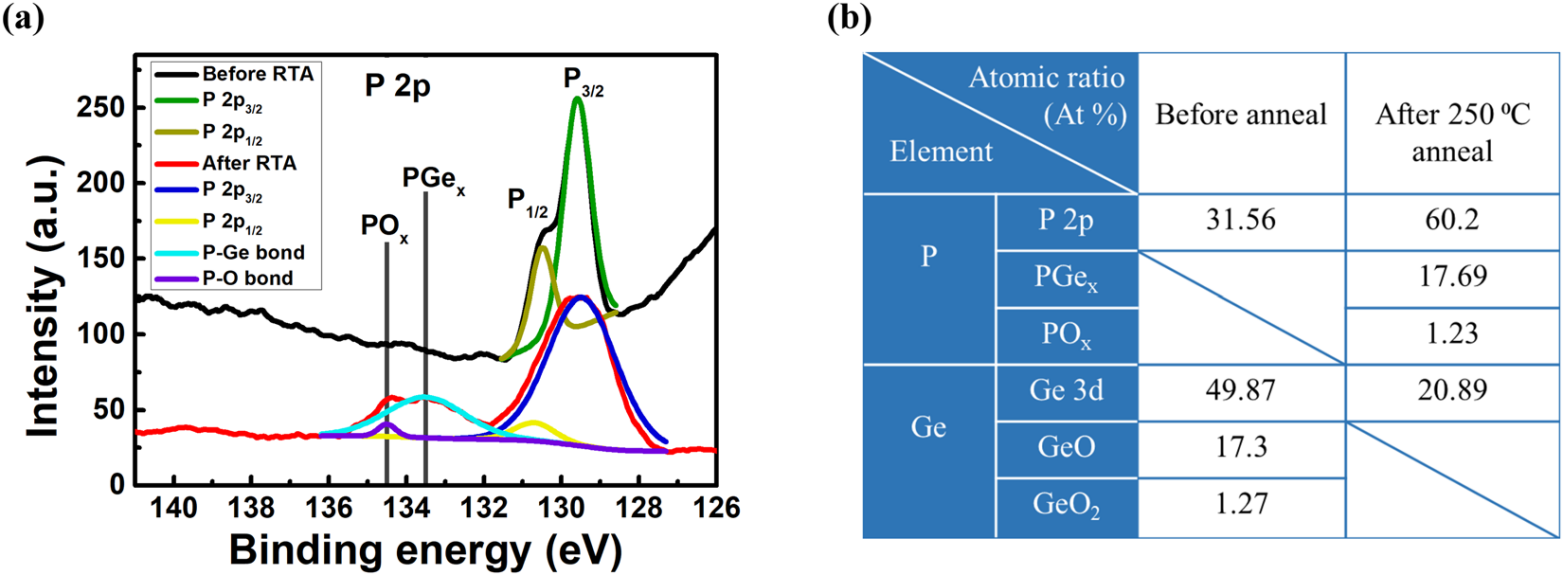
(**a**) XPS results of BP with 1.5 nm Ge on top of it before and after RTA. After RTA, the peak of P-Ge bond reveals. (**b**) The atomic ratio (At %) of each bond before and after RTA. It is clear that after RTA, the At % of BP increases while Ge decreases. After annealing, PGe_x_ forms and is classified into P signals. In addition, the doping of Ge into BP enables more P signals to be detected.

The reaction between BP and Ge after annealing is characterized by X-ray photoelectron spectroscopy (XPS). XPS spectra are taken of the P 2p, Ge 3d, C 1 s core levels using a monochromatic Al Kα X-ray as the source. Due to the large spot size of X-ray (diameter of 400 μm), the measured peak intensity will be close to the background noise if the amount of BP flakes are few. To maintain the peak intensity, bulk BP is directly exfoliated onto a copper tape adhered on a silicon substrate. The conductive copper tape also help avoid the charging effect during XPS characterization. After exfoliation, a thin layer (1.5 nm) of Ge was deposited onto the sample. Note that the thickness of Ge needs to be thin enough or the X-ray cannot penetrate through Ge atoms and reach Ge-BP interfaces. Totally two samples are prepared, with one undergoing the RTA process and the other does not. The conditions of RTA is the same as that used at FET fabrication: 250 °C for 1 minute in nitrogen environment. The P 2p XPS results of these two samples are shown and compared in Fig. 6a. In the sample without RTA, the spectrum shows two peaks: P 2p_3/2_ (129.6 eV), P 2p_1/2_ (130.5 eV), which is due to the effect of spin-orbit splitting at 2p level^44^. After annealing, two additional peaks located at 133.5 and 134.5 eV emerge. The peak at 134.5 eV is related to P-O bond, while the peak at 133.5 eV is assigned to P-Ge bond^45–47^. The origin of the P-O bond might be due to the moisture on the copper tape reacts with BP during RTA process. The atomic percentage (At %) of each bond is also calculated by the peak area divided by the atomic sensitive factor. The comparison of At % for all bonds before and after anneal is listed in Fig. 6b. Before RTA process, the total At % of P and Ge is 31.56% and 69.44%, respectively. The higher At % of Ge is probably due to the fact that Ge is deposited on top of BP, resulting in more signal of Ge detected by the XPS instrument. After RTA process, the At % of P increases to 79.11% and Ge decreases to 20.89%. As we can see, the major enhancement of signal of P lies in the formation of P-Ge bond (17.69 At %), while P-O bond contributes little (1.23 At %). Moreover, the doping of Ge into BP also enables more BP signals to be detected, thus leading to the increase of atomic percentage. Hence, we are able to confirm that germanium successfully dopes into BP and a metallic PGe_x_ compound is formed after RTA.

## Conclusion

In summary, we successfully fabricate BP transistors with metallic PGe_x_ contacts by RTA treatment. The electrical results before and after RTA process is characterized in a vacuum chamber. After RTA, the mobility can be improved by beyond 25 times and the highest mobility of 227 cm^2^V^−1^s^−1^ is extracted. Moreover, the contact resistance after RTA is also obtained using TLM with an ultra-low R_c_ of 0.365 kΩ∙μm, which is the lowest value reported in the back gate BP transistors without degradation of I_ON_/I_OFF_ ratio. In addition, the I-V curve remains linear for PGe_x_ contact at 80 K compared to Ti contact. TLM results of PGe_x_ contact at various temperatures demonstrate for the first time a metallic contact is achieved in BP transistors. Finally, the chemical reaction of forming bonds between Ge and BP is supported by XPS characterization. The XPS results show that after RTA, a peak of P-Ge bond emerges and the detected BP signal increases, which further verifies the doping of Ge into BP and the formation of PGe_x_.

## Methods

Multilayer BP is mechanically exfoliated from bulk BP grown by Prof. P. D. Ye from Purdue University. The transferred residue is cleaned by acetone. Raman spectroscopy is characterized by a 630 nm-green light laser. Bi-layer PMMA (PMMA 495 A4/ PMMA 950 A4) is spin-coated on the surface of the sample for S/D pattern definition by EBL process (JEOL JSM-7001F). Development is performed in 1:3 MIBK: IPA (Methylisobutyl ketone: Isopropanol) for 60 s. S/D metal is deposited by E-gun evaporator under 10^−6^ Torr. Electrical measurement is carried out by Keysight B1500A.

## Electronic supplementary material

Supplementary Information
