# Supplementary material for: Germanium-doped Metallic Ohmic Contacts in Black Phosphorus Field-Effect Transistors with Ultra-low Contact Resistance
Source: Sci Rep. 2017 Dec 4;7:16857. doi: 10.1038/s41598-017-16845-w (PMC5714961; doi:10.1038/s41598-017-16845-w)
Supplement: Supplementary file 1 — Supplementary Information [file 41598_2017_16845_MOESM1_ESM.pdf]

Supplementary information:

Germanium-doped Metallic Ohmic Contacts in Black Phosphorus Field-Effect Transistors with Ultra-low Contact Resistance

Hsun-Ming Chang[†], Adam Charnas[c], Peide D. Ye[c], Yu-Ming Lin[*,¶], Chih-I Wu[†,b], and Chao-Hsin Wu[*,†,a]

[†]Graduate Institute of Photonics and Optoelectronics, National Taiwan University, No. 1, Sec. 4, Roosevelt Road, Taipei 10617. Taiwan (R.O.C.).

[a]Graduate Institute of Electronics Engineering, National Taiwan University, No. 1, Sec. 4, Roosevelt Road, Taipei 10617, Taiwan (R.O.C.).

[¶]Taiwan Semiconductor Manufacturing Company, No. 8, Li-Hsin Rd. 6, Hsinchu Science Park, Hsinchu City 300, Taiwan (R.O.C.)

[b]Industrial Technology Research Institute.
195, Sec. 4, Chung Hsing Rd., Chutung, Hsinchu City 31040, Taiwan (R.O.C.)

[c]School of Electrical and Computer Engineering and Birck Nanotechnology Center, Purdue University, West Lafayette, Indiana 47907, United States.

Email: ymlinw@tsmc.com, chaohsinwu@ntu.edu.tw

1. **Improvement of hysteresis in transfer curve after RTA**

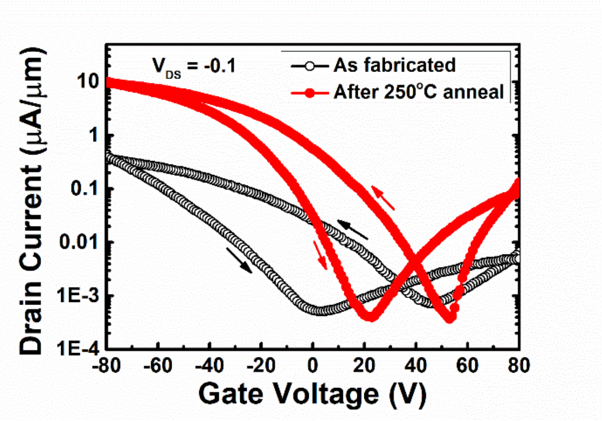

Figure S1. Transfer curve of a Ge contact BP transistor before and after 250 ^{0} C rapid thermal annealing. The arrow indicates the sweep direction of gate voltage. We can observe that the hysteresis reduces after RTA treatment.

2. **Annealing effect on Ti contact BP FET**

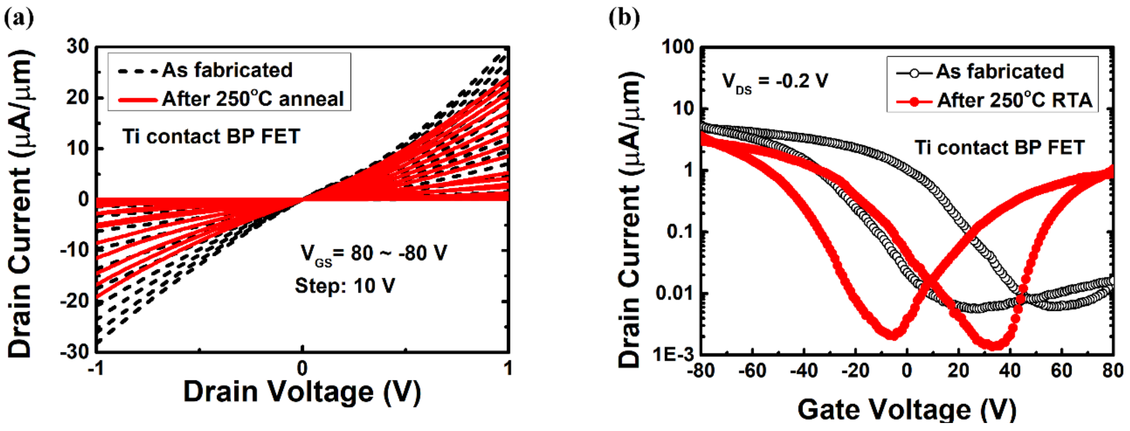

Figure S2 (a) Output characteristics of a Ti contact BP transistor before and after 250 ^{0} C anneal. As the results shown, there is no improvement in drain current after RTA treatment. (b) Transfer curve of the same device before and after RTA treatment. It is clear that a left-shift V_{TH} is observed, resulting in degradation of hole current and enhancement of electron current, respectively.

3. Detailed derivation of intrinsic mobility

The derivation of the intrinsic mobility μ_{int} starts from the fomula of output characteristics in linear region:

$$I_{DS} = \mu_{int} C_{ox} \frac{W}{L} (V_{GS} - V_{TH} - I_{DS} R_c)(V_{DS} - 2 I_{DS} R_c).$$

From which we can derive the transconductance G_m by:

$$G_m = \frac{dI_{DS}}{dV_{GS}} = \frac{W}{L} \mu_{int} C_{ox} \left[V_{DS} - V_{DS} \frac{dI_{DS}}{dV_{GS}} R_c - V_{DS} I_{DS} \frac{dR_c}{dV_{GS}} - 2 \frac{dI_{DS}}{dV_{GS}} R_c (V_{GS} - V_{TH}) - \right.$$

$$\left. 2 I_{DS} \frac{dR_c}{dV_{GS}} (V_{GS} - V_{TH}) - 2 I_{DS} R_c + 4 I_D \frac{dI_{DS}}{dV_{GS}} R_c^2 + 4 R_c \frac{dR_c}{dV_{GS}} I_{DS}^2 \right].$$

Since R_c is dependent on V_{GS}, we fit R_c vs. V_{GS} by polynomial function: $R_c = a + b(V_{GS} - V_{TH}) + c(V_{GS} - V_{TH})^2 + d(V_{GS} - V_{TH})^3$, where the fitting parameters are extracted as:

$$a = 3.79799$$

$$b = 0.11294$$

$$c = 0.00135$$

$$d = 5.79201 \times 10^{-6}$$

The fitting results are shown below and in Figure S3. After obtaining dR_c/dV_G, we can extract μ_{int} by G_m.

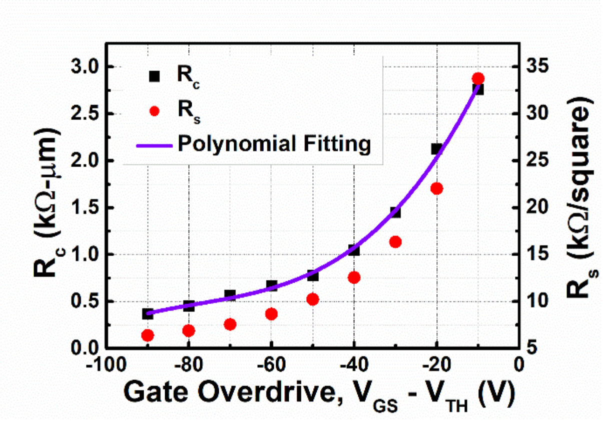

Figure S3. R_c and R_s vs. gate overdrive, with R_c fitting by polynomial function.

4. Power law fitting of ρ_c vs. T

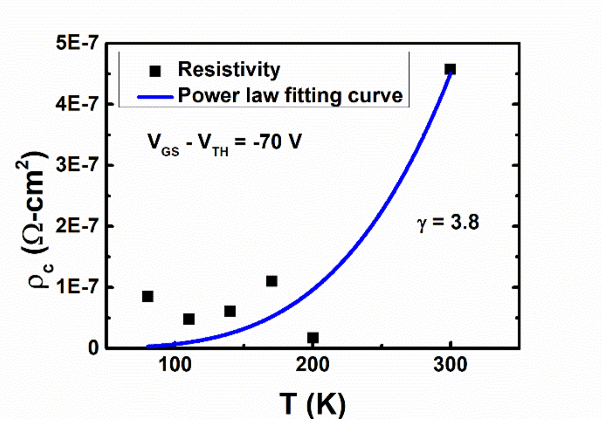

Figure S4. Contact resistivity ρ_c is plotted with various T and fitted by power law: $\rho_c(T) \propto T^{\gamma}$. The extracted γ of +3.8 describes the metallic properties of the PGe_x contact.
